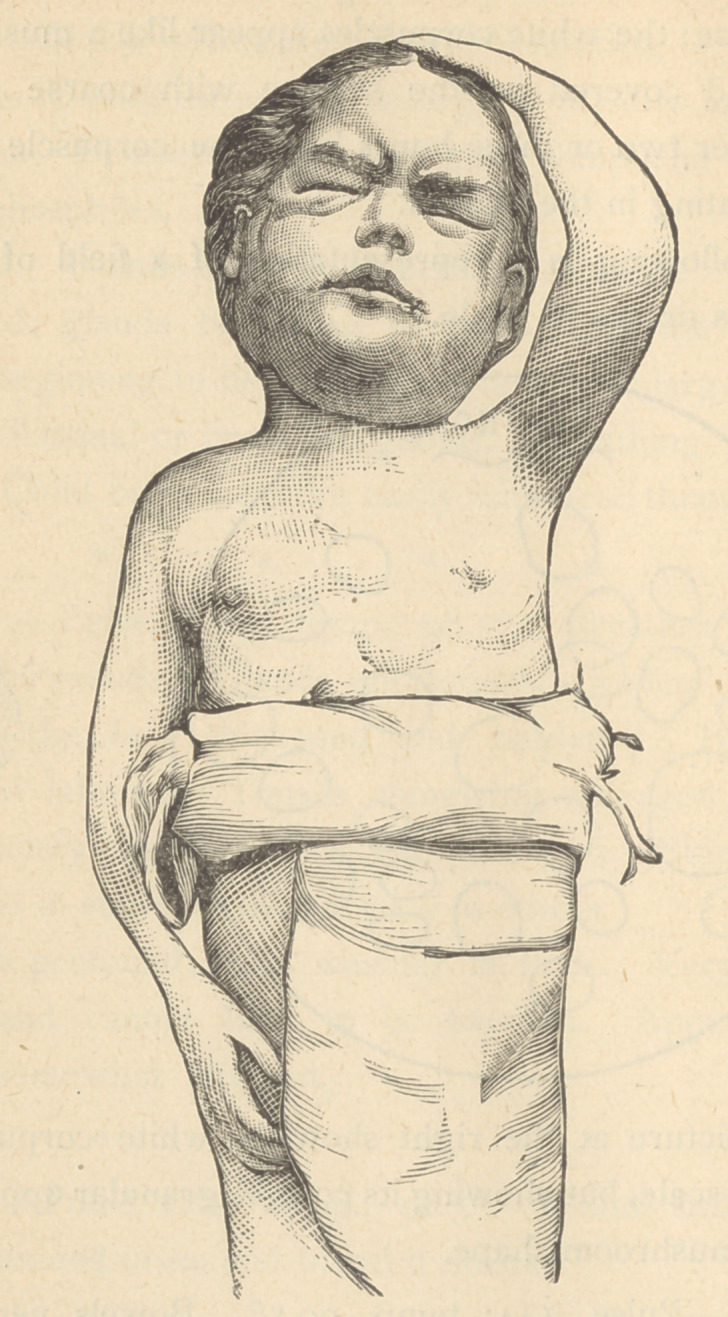# Lymphadenoma

**Published:** 1886-06

**Authors:** J. W. Nelson

**Affiliations:** Winnetka, Illinois


A CASE OF LYMPHADENOMA. *By* J. W. NELSON, M.S., M.D., *Winnetka, Illinois.*

The disease commonly known as Hodgkins' disease, also known as lymphadenoma, pseudo-leueocythæmia, etc., although a comparatively rare disease and not often met with by the general practitioner, yet occurs with sufficient frequency to merit careful study, inasmuch as its etiology is so little known, its treatment uniformly ineffective and its results inevitably fatal.

Up to the present time those who have studied the disease have been unable to go beyond a certain point, nor will the cause of lymphadenoma, in all probability, ever be discovered until the origin of the red and white blood corpuscles has been found, and also the relation between the two established.

The object of this paper, however, is not a discussion of the various theories concerning the cause of lymphadenoma, but simply to record the history of a case now under the care of the writer, and to draw conclusions from it. However, a short review of the disease and its treatment may not be out of place.

Lymphadenoma manifests itself chiefly in the lymphatic system. There is an enlargement of the lymphatic glands by a deposit of adenoid tissue, also a deposit of the same kind along the course of the lymphatics and often in regions where there are no glands, but simply minute lymph channels. These enlargements vary in size and density, the more newly formed being less dense than than those of older growth. They vary in size from that of a pea to a hen's egg and even larger, a few have been recorded weighing two or three pounds. The posterior glands are usually the first affected, though not invariably, the affection spreading next to the other glands of the neck, then to the axilla, mediastium mesentery and inguinal region.

In some cases the enlargement is confined to only one or two groups of glands, but the tendency is to involve the whole lymphatic system. Apart from the phenomena exhibited in the lymphatic system, the most interesting features of the disease are to be seen in an examination of the blood. Excessive anæmia is always present in lymphadenoma. But more will be said with regard to the conditions of the blood further on.

In this disease, as in other stubborn affections, the whole pharmacopœia has been searched for remedies with little or no success. Nearly every drug classed as an alterative has been used, the best results being obtained from the use of mercury, iodine, iron, phosphorus and arsenic in their various forms. Of all these, the best results are claimed from the use of arsenic in the form of the liquor potassii arsenitis in large doses.

Extirpation of the enlarged glands has been resorted to with but little benefit, the glands in other localities invariably enlarging.

The following case came under my care Sept. 16, 1885 :*

Mary C.; child, 6 years old; lives with her parents at — W. 22d street, Chicago. Locality in close proximity to south branch of Chicago river, low quarter of the city and badly drained. Parents, Irish. Father, day laborer, very intemperate in habits. Mother apparently healthy. No history of scrofula, tuberculosis, syphilis or lymphadenoma in the family. Found the child markedly anæmic, poorly nourished, and with hard tumors in the lymphatic glands and in the course of the lymph channels of the right side of the head and face. The cervical and submaxillary glands of the right side especially large and hard. Right eye closed by hardening and thickening of both lids. Especial thickening over the supercilliary ridges and in line of the temporal artery. Counted forty distinct enlarged glands, beside deposits elsewhere, the tumors ranging in size from that of a pea to a small hen's egg, the largest being in the right submaxillary region. On palpation could find no enlargement of spleen or liver. Percussion gave dullness in the median line over the thorax. Auscultation gave heart sounds normal, but rapid; mucous râles over the larger bronchi. From the constant cough, and dullness over the median line of the thorax, concluded that the mediastinal or bronchial glands were involved. Temperature, 103°; pulse, 168; respiration, 28. Rapidity of pulse due to excitement. Skin dry and scaly and covered with small sudaminous vesicles. To reduce the temperature, gave

| | | |
|---|---|---|
| R.—Potassii nitratis | 6 | c. c. |
| Spts. ætheris nit | 16 | " |
| Liq. ammoni acetat | 16 | " |
| Aquæ, q. s. ad | 128 | " |

M. f℥ss. every two hours.

*The first enlargement was noticed in May, 1885.

Sept. 17.—Child apparently better; could not take temperature on account of broken thermometer. Pulse, 140. Discontinued the above prescription and gave the following:

℞.—Liquor. potassii arsenitis......................f℈iv.

Syr. simp., q. s. ad..............................f℥i.

M. f℈i twice a day.

Sept. 22.—Child apparently better, sitting up; no appreciable fever. Pulse, 126. Some of the glands seem slightly softened and reduced in size, especially those in axillæ and groins.

Sept. 24.—Child a little stupid. Temp., 98.8°; pulse, 130, runs up at the least excitement. Appetite good. Marked decrease in size of axillary glands. Increased the amount of Fowler's solution by giving three doses per day. Also gave the following:

℞.—Tinct. ferri chloridi..........................f℈iii.

Syr. simp., q. s. ad..............................f℥iv.

M. f℈i t. i. d. after meals.

Ordered the mother to give the child a thorough bath every day, to use massage over the enlarged glands and to lay the child naked for at least an hour in the day in the direct rays of the sun.

Sept. 26.—Pulse, 120; temp., 98°. Skin dry and still covered with small vesicles. Glands much reduced in size.

Sept. 28.—Pulse, 92; temp., 98.4°. Child bright, appetite good, still farther reduction in the size of the glands. Increased the dose of Fowler's solution to 10 drops three times a day.

Oct. 3.—Pulse, 150; temp., 98.5°. Appetite good.

Glands smaller than when last seen. Fowler's solution beginning to show systemic effects. Discontinued it.

Oct. 5.—Pulse, 160; temp., 96.5°. Effects of the arsenic cease to show; gave :

℞—Acidi Phos. Dil.............................fʒiv

Syr. Hypophos. Comp.......................f℥ii

Syr. Simp., q. s. ad..............................f℥iv

M. fʒi t. i. d. before meals.

Oct. 8.—Pulse, 144; temp., 98.5°. Tongue clean. Glands at a stand-still. Renewed Fowler's solution—10 drop doses t. i. d.

Oct. 12.—Pulse, 152; temp., 98°. Bowels quite loose. Discontinued the arsenic; renewed the phosphoric acid mixture and gave Emulsion I of Dispensary Formula.

Oct. 17.—Pulse, 132; temp., 98.5°. Renewed the arsenic. Glands decreasing slightly.

Oct. 20.—Pulse, 135; temp., 98.5°. Glands in axillæ and groins reduced almost to natural size. In left groin one gland still about the size of a chestnut, also one of the same size in the right axilla. Patient able to see out of the right eye that was formerly closed. Glands in submaxillary region still remain hard.

Oct. 23. On examining child's mouth found three badly decayed teeth, and thinking they might be a source of irritation ordered them extracted.

Oct 26.—Mother took the child to a dentist and had two teeth in the left lower jaw extracted. Child again beginning to show symptoms of arsenic poisoning. Discontinued Fowler's solution and renewed the phosphoric acid, etc.

Nov. 1.—Found the glands rapidly increasing in size; glands in left submaxillary region near where the teeth were extracted much enlarged. Eyelids of left eye becoming thickened. Renewed Fowler's solution.

Nov. 5.—Glands increasing with great rapidity in spite of the arsenic. Discontinued it and gave compound syrup of the hypophosphites.

Nov. 12. Glands as large as before treatment. Left eye half closed; glands beginning to press upon the trachea. Sternum beginning to be pressed forward by enlargement of either mediastinal or bronchial glands. Breathing growing croupy. Child breathes more easily with head thrown back.

Dec. 2.—Pulse, 150; temp., 98.5°. Breathes entirely through the mouth. Glands enormously swollen. All the tissues of the face thickened and hardened. Breathing croupy and labored. Tonsils somewhat enlarged. Right eye completely closed and left eye nearly so. Skin almost transparent in some parts and scaly in others.

Anæmia profound; child wasting in flesh. Very hyperæsthetic and cannot bear to be handled. Appetite fair. Hearing somewhat affected.

Dec. 9.—Pulse, 120; temp., 98.5°. Glands still enlarging. Examined urine and found it normal.

Dec. 22.—Took child to South Side Dispensary. Dr. Lester Curtis examined the blood microscopically; found it pale and watery; coagulates rapidly. Relative quantity of red blood corpuscles greatly diminished. Slight increase, if any, white corpuscles. Red corpuscles very irregular in size, many of them not more than one third or one fourth natural size; the white corpuscles appear like a mushroom in shape and covered on the surface with coarse granules which after two or three hours leave the corpuscle and are found floating in the plasma.

The following is a representation of a field of the red corpuscles drawn to scale.

The picture at the right shows a white corpuscle not drawn to scale, but showing its coarse, granular appearance, also its mushroom shape.

Jan. 4.—Pulse, 144; temp., 99.5°. Bowels very loose. Glands still enlarging. Left eye nearly closed. Sterum somewhat protruding.

Jan. 12.—Pulse, 108; temp., 99.5°. Increase in difficulty of breathing. Eyes closed. Hearing very dull. Appetite poor.

Jan. 25.—Took child to a gallery and obtained a photograph.

Child able to see a little out of left eye. Breathing difficult for the last two days.

From the foregoing data very little that is new can be deduced, unless it be the highly granular appearance of the white corpuscles and the great irregularity in size of the red.

With regard to the treatment, there is fair ground for belief that if a case of lymphadenoma could be diagnosticated in its incipient stage, a free use of arsenic as an alterative, and iron to relieve the anæmia, would result in a permanent cure.

## Figures and Tables

**Figure f1:**
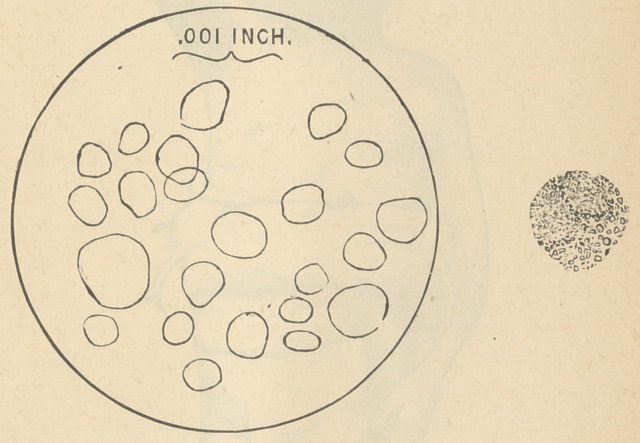


**Figure f2:**